# Physics-Informed Neural Networks for Depth-Dependent Constitutive Relationships of Gradient Nanostructured 316L Stainless Steel

**DOI:** 10.3390/ma18153532

**Published:** 2025-07-28

**Authors:** Huashu Li, Yang Cheng, Zheheng Wang, Xiaogui Wang

**Affiliations:** College of Mechanical Engineering, Zhejiang University of Technology, Hangzhou 310032, China; 211122020004@zjut.edu.cn (H.L.); 111122020022@zjut.edu.cn (Y.C.); www845475039@foxmail.com (Z.W.)

**Keywords:** physics-informed neural networks, gradient structures, elastic–plastic constitutive relationships, strain hardening parameter, transfer learning

## Abstract

The structural units with different characteristic scales in gradient nanostructured (GS) 316L stainless steel act synergistically to achieve the matching of strength and plasticity, and the intrinsic plasticity of nanoscale and ultrafine grains is fully demonstrated. The macroscopic stress–strain responses of each material unit in the GS surface layer can be measured directly by tension or compression tests on microspecimens. However, the experimental results based on microspecimens do not reflect either the extraordinary strengthening effect caused by non-uniform deformation or the intrinsic plasticity of nanoscale and ultrafine grains. In this paper, a method for constructing depth-dependent constitutive relationships of GS materials was proposed, which combines strain hardening parameter (hardness) with physics-informed neural networks (PINNs). First, the microhardness distribution on the specimen cross-sections was measured after stretching to different strains, and the hardness–strain–force test data were used to construct the depth-dependent PINNs model for the true strain–hardness relationship (PINNs_εH). Hardness–strain–force test data from specimens with uniform coarse grains were used to pre-train the PINNs model for hardness and true stress (PINNs_Hσ), on the basis of which the depth-dependent PINNs_Hσ model for GS materials was constructed by transfer learning. The PINNs_εσ model, which characterizes the depth-dependent constitutive relationships of GS materials, was then constructed using hardness as an intermediate variable. Finally, the accuracy and validation of the PINNs_εσ model were verified by a three-point flexure test and finite element simulation. The modeling method proposed in this study can be used to determine the position-dependent constitutive relationships of heterogeneous materials.

## 1. Introduction

Strength refers to the ability of a material or component to resist failure when subjected to external loads, and ductility denotes the maximum permanent deformation that a material or component can withstand before failure. In engineering applications such as machinery, aviation, and transportation, strength and ductility are two critical indicators. The metallic materials typically struggle to achieve both high strength and high ductility due to their mutually exclusive nature [1,2,3,4,5,6]. However, the synergy of strength and ductility has been achieved by constructing a gradient nanostructured (GS) surface layer on bulk metallic materials by surface mechanical attrition treatment (SMAT) [7,8,9]. SMAT induces gradient nanostructures through severe plastic deformation, which involves dislocation slip and twinning mechanisms. To date, this method has been successfully applied to many pure metals such as Cu [8], Fe [10], Ni [11], and Ti [12], and alloys such as stainless steel [13,14,15,16,17], titanium alloys [18], and nickel-based alloys [19].

The size of structural units in GS materials exhibits gradient changes in space, increasing continuously from the nanoscale to the macroscale [8]. Due to the non-uniform mechanical properties of different structural units along the gradient direction, the GS surface layer should be considered as heterogeneous. To study the heterogeneous mechanical properties, the GS surface layer can be considered as a collection of representative volume elements (RVE). Each RVE contains a sufficient number of discrete structural units to yield uniform macroscopic mechanical properties after statistical averaging. The preparation of specimens for macroscopic mechanical property testing, as well as the selection of testing equipment, should be consistent with the dimensions of the RVE. Due to the extremely small dimension of the RVE along the gradient direction, the preparation of miniature specimens for macroscopic mechanical property testing is very challenging.

To address this challenge, researchers have used advanced experimental techniques, such as the instrumented micro/nano indentation experiment [20] and micropillar compression tests, with micropillar specimens prepared using Focused Ion Beam (FIB) technology [21], to study the mechanical properties of materials. However, these techniques still have certain limitations. Instrumented micro/nano indentation experiments are capable of measuring material strength but cannot provide detailed and accurate stress–strain curves. The micropillar compression test allows for the direct measurement of the macroscopic stress–strain response, but the derived stress–strain curves typically show higher values than those of macroscopic specimens, and the correlation between these results and those obtained from bulk specimen tests remains a subject of debate. In addition, miniature specimens can be machined for tensile testing to determine the stress–strain response. However, these specimens exhibit necking at very small strains (~5%) due to strain localization, resulting in an inability to obtain the intrinsic ductility of the material [22]. In summary, it remains a challenge to accurately determine the macroscopic stress–strain responses of any RVE within the GS surface layer using direct measurement techniques.

In addition to experimental studies, constitutive models based on microphysical mechanisms [23,24,25,26,27,28,29,30] have been developed in order to account for the influence of microstructural features of the materials (e.g., grain boundaries, twins, and dislocations, etc.) on their mechanical properties. Li et al. [23,24,25] systematically investigated the influence of microstructure on the stress state and additional work hardening of a gradient nanostructured interstitial-free (GS-IF) steel subjected to axial tension. A theoretical model based on microscopic physical mechanisms was then proposed, in which the influence of grain size on strength was characterized by the Hall–Petch relationship and the deformation incompatibility caused by gradient structures was characterized by geometrically necessary dislocations. Lyu et al. [26] investigated the effect of gradient nanostructures on the strength and ductility of GS-IF steel based on a multi-scale dislocation model combined with strain/stress gradient plasticity theory. Based on the assumption that all slip impedance parameters obey the Hall–Petch relationship, a crystal plasticity plane strain model was developed for GS Cu, and the model successfully reproduced the gradient distributions of the stress and strain fields [27]. Zhu et al. [28] established a microstructure-based constitutive model for GS 304 stainless steels, incorporating phase transformation and dislocation evolution. Zhang et al. [29] developed a constitutive framework for hierarchical dual-phase gradient alloys, addressing stress partitioning between martensite and austenite phases. Jin et al. [30] quantified the effects of grain size gradient magnitude and subsurface layer thickness on mechanical properties through length-scale-dependent constitutive modeling. Although these models are better at reproducing the whole macroscopic mechanical properties of the GS materials, they are not able to give the macroscopic mechanical properties of any structural units in the GS surface layer.

Numerical simulation [31,32,33,34,35,36] is also an effective way of investigating the intrinsic mechanical behavior of GS materials. A crystal plasticity model incorporating dislocation slip and deformation twinning was developed to describe the tensile response of Cu [32] and TWIP steels [33] with gradient varying grain sizes. Molecular dynamics simulation was used to study the mechanical behavior and phase transformations in GS NiTi shape memory alloys [35], and the tensile properties and deformation mechanisms of GS copper [36]. It should be noted that both mesoscale crystal plasticity finite element simulations and nanoscale molecular dynamics simulations, while helpful in deepening the understanding of the relationship between microstructure and macroscopic mechanical properties, are unable to provide the macroscopic elastic–plastic constitutive relationships required for finite element simulations of engineered components made of GS materials.

In the case of insufficient or missing experimental data, Physics-Informed Neural Networks (PINNs) are a good choice for the study of constitutive relationships. PINNs integrate data-driven approaches with physical laws, allowing for effective exploration of the constitutive relationships of complex materials [37,38,39]. By incorporating differential equations, initial conditions, and boundary conditions into the loss function, PINNs embed physical laws directly into the model, effectively enforcing physical constraints [40]. Compared to traditional deep learning methods, PINNs achieve robust predictive capabilities and improved generalization to complex and noisy datasets, thereby reducing the reliance on extensive experimental data. PINNs are especially suitable for scenarios where there is little or no experimental information. In addition, PINNs use automatic differentiation (AD) technology, which computes values through chain rules rather than symbolic derivatives, allowing efficient handling of high-dimensional problems [41]. These features give PINNs the potential to model the mechanical behavior of materials with a gradient structure.

The aim of this paper is to obtain depth-dependent elastic–plastic constitutive relationships of GS 316L stainless steel. First, the cross-sectional hardness distributions and strain-load data of GS and uniform coarse-grained (CG) 316L specimens were obtained after experiencing different tensile strains, from which the depth-dependent PINNs_εH model was constructed. Then, based on the positive correlation between hardness and strain (stress), the test data of hardness, strain, and stress of the CG specimens were used to pre-train the depth-dependent PINNs_Hσ model. By equivalent static conditions rather than stress data-driven, a PINNs_Hσ model for GS 316L stainless steel was constructed by transfer learning from the pre-trained model. Considering the physical nature of no strain-hardening effect during elastic deformation and no apparent yield limit, the PINNs_εσ model of the depth-dependent elastic–plastic constitutive relationships of GS 316L stainless steel was constructed with hardness as an intermediate variable. The applicability of the model covers the entire range from the elastic stage to the uniform plastic deformation stage of the material. Finally, the experimental and numerical results of the compressive load and mid-span deflection of the three-point bending specimen are in good agreement, validating the methodology and confirming the accuracy of the PINNs_εσ model. The constitutive model construction method proposed in this study is independent of the specific chemical composition of the material. Its universality stems from the fundamental requirement to characterize the material’s mechanical property gradient and its strain hardening capability using hardness. This provides a theoretical foundation for extending this approach to constitutive relationship research in other graded metallic material systems.

## 2. Quasi-Static Tensile Test and Hardness Measurement

**Figure 1:**
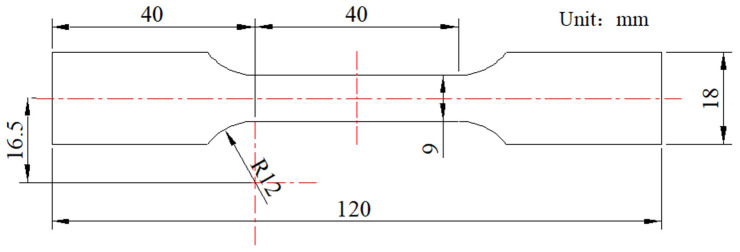
Tensile specimen with a thickness of 7.0 mm.

Quasi-static uniaxial tensile tests of 316L stainless steel were conducted at room temperature using an Instron 3369 universal material testing machine with a load cell capacity of 50 kN. An extensometer with a range of ±40% was used to measure the axial strain. The tensile specimens with rectangular cross-sections, as shown in Figure 1, were used. Two types of tensile specimens were used: one was the original CG specimen with uniform grain size, and the other was a sandwich specimen with GS surface layers on both sides and a CG matrix in the middle. The sandwich specimens were prepared using a double-sided symmetrical rolling process. The two GS surface layers of the sandwich specimen are symmetrical about its midplane.

**Figure 2:**
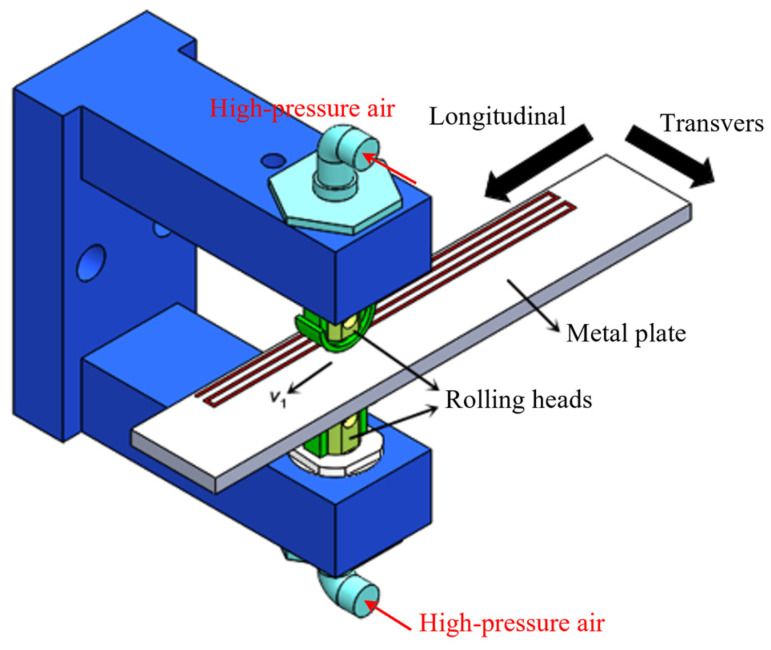
A visualization of the SMRT equipment [42].

A double-sided surface mechanical rolling treatment (D-SMRT) device was employed to fabricate GS surface layers on both sides of CG 316L stainless steel plates [42]. As shown in Figure 2, the D-SMRT device is equipped with two identical rolling heads, each containing a 6 mm diameter WC/Co cemented carbide ball and driven by high-pressure air. The high-pressure air passes through a pressure conversion assembly, which converts the air pressure (*P*) into a force (*F*) exerted by the WC/Co alloy balls on the surface of the specimen. There is a linear relationship between *F* and *P*, *F* = −11.512 + 484.34*P*, where *F* is in *N* and *P* is in MPa. To obtain a thicker gradient-structured surface layer and a more refined microstructure, five rolling passes were conducted. The air pressure applied to the rolling head was progressively increased from 0.4 MPa to 1.6 MPa with a stepwise increment of 0.3 MPa per pass. During the processing, the longitudinal velocity of the adaptive D-SMRT tool was 108 mm/s, while the transverse feed after the adaptive D-SMRT was 0.04 mm. Clean oil lubricant was used to lubricate and cool WC/Co cermet balls and specimens during the process.

**Figure 3:**
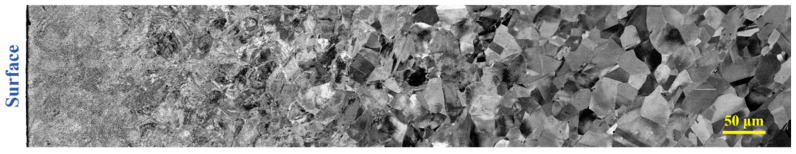
Cross-sectional microstructures of GS-316L stainless steel.

Figure 3 presents the cross-sectional microstructure of the sandwich specimen of GS-316L stainless steel. The GS surface layer comprises structural units with distinct mechanical properties, including grains, twins, martensite, and austenite [43]. The size of these structural units exhibits a gradient variation along the depth direction, ranging from the nanoscale to the macroscale. The GS surface layer possesses a thickness of ~1 mm. Given that the characteristic size of the structural units is 2–5 orders of magnitude smaller than this thickness, the macroscale mechanical properties can be considered continuously graded along the depth direction.

**Figure 4:**
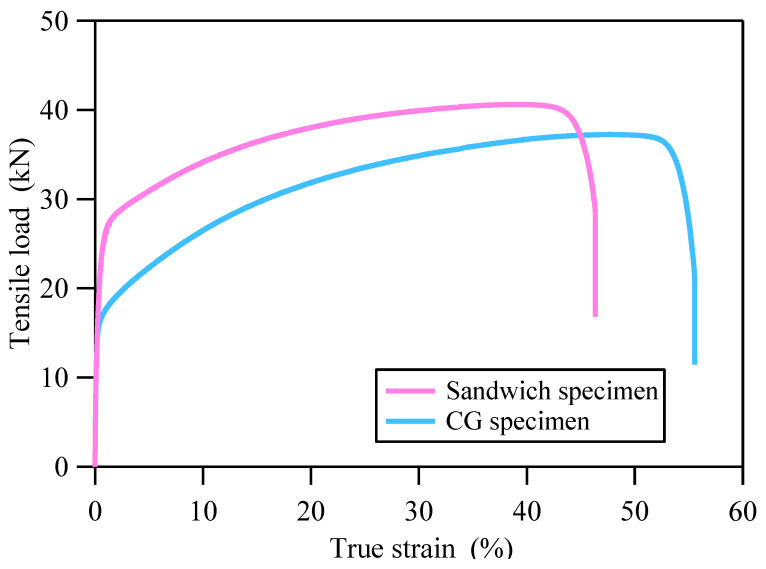
The curves of tensile load and true strain of CG and sandwich specimens.

The curves of axial tensile load and true strain for both types of specimens are given in Figure 4. The uniform elongation and maximum tensile load of the CG specimen were 47.9% and 37.2 kN, respectively. The uniform elongation and maximum tensile load of the sandwich specimen were 39.2% and 40.6 kN, respectively. It is obvious that the CG specimen has greater uniform elongation, while the sandwich specimen has a higher maximum tensile load.

Eleven engineering strain values, 2%, 5%, 7.5%, 10%, 15%, 20%, 22.5%, 25%, 30%, 35%, and 39%, were selected within the uniform elongation range of the sandwich specimens. The specimens were stretched to the given strains and then unloaded. A series of samples for measuring cross-sectional hardness were prepared using the gauge segment of the tensile specimens subjected to different plastic deformations. A Hvs-1000M microhardness tester with an indentation load of 100 gf and a dwell time of 10 s was used to measure the hardness. After axial tension, the transverse dimensions of the specimens decreased correspondingly. Therefore, for hardness measurement samples subjected to different axial tensile strains, the distance between hardness measurement points with the same apparent depth and the surface is different in the undeformed state. Based on the assumption of plastic incompressibility for metallic materials, the apparent depth $d_{0}$ of the hardness measurement point can be converted to the initial depth d by $d=d_{0}\sqrt{1+\varepsilon_{e}}$, where $\varepsilon_{e}$ represents the axial engineering strain.

**Figure 5:**
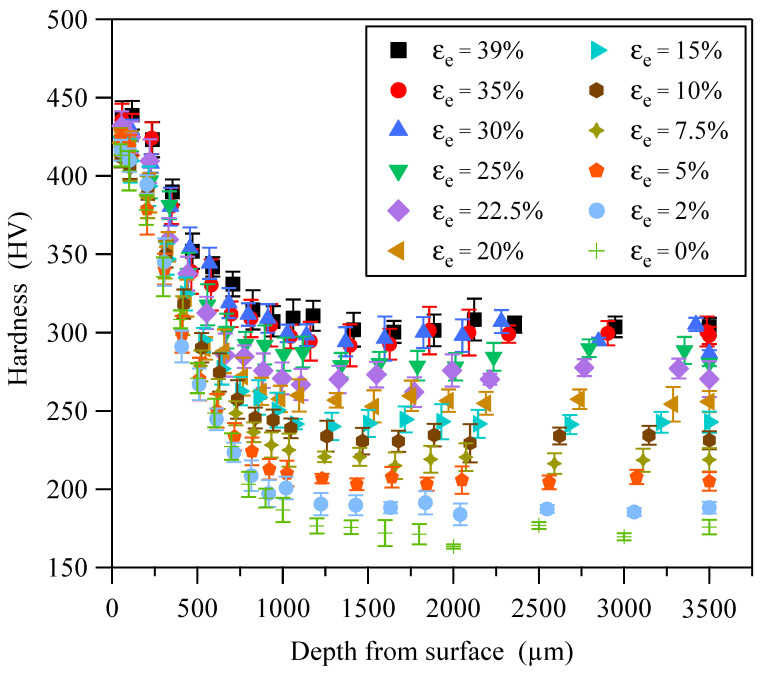
Cross-sectional hardness distributions of sandwich specimens subjected to different tensile strains.

Considering that hardness gradually changes along the thickness direction of the GS surface layer, the measurement locations should be selected reasonably according to the microhardness change rate. There should be at least five valid data points at the same measurement depth. The measurement results of initial depth, engineering strain, and hardness are shown in Figure 5. It can be seen that the hardness decreases with increasing depth at the same engineering strain and stabilizes at a certain depth (greater than 1200 µm). At the same depth from top surface, the hardness increases with the increase in engineering strain, indicating that hardness has the capability to characterize strain hardening of metallic materials.

## 3. Depth-Dependent PINNs_εH Model

**Figure 6:**
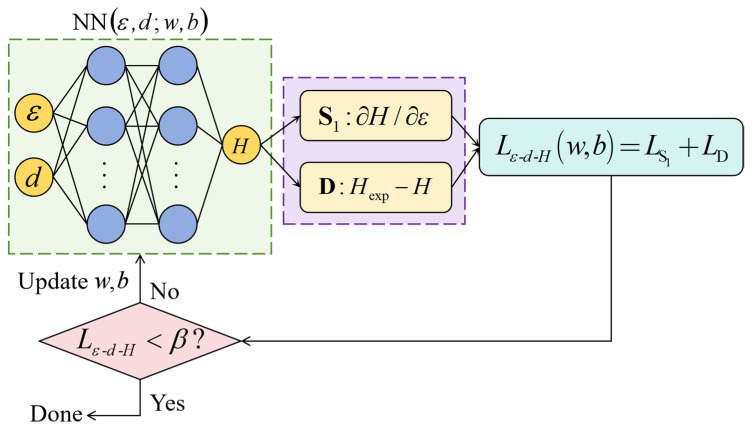
Constructing a depth-dependent PINNs_εH Model for true strain and hardness.

It can be seen from the measured hardness shown in Figure 5 that there is a certain degree of dispersion in the cross-sectional hardness. The dispersion is related to some factors, such as the inhomogeneity of the material microstructure, the precision of the hardness tester, and the depth deviation of measurement locations. In addition, in the three-dimensional space composed of depth, engineering strain, and hardness, the values of measured hardness are discrete data points, so it is impossible to directly obtain hardness corresponding to any depth and engineering strain. To address this issue, the powerful generalization capability of PINNs can be utilized to construct a relationship model among depth, true strain, and hardness. The model based on PINNs not only eliminates the dispersion of experimental data but also provides a vast amount of data for subsequent study of constitutive relationships. A fully connected feedforward neural network was employed for PINNs_εH modeling, as illustrated in Figure 6. The true strain *ε* and initial depth *d* are the input features, and hardness *H* is the output label.

The loss function of PINNs can force the neural network to satisfy physical laws by combining physical constraints. Therefore, compared to traditional neural networks trained solely on data, PINNs exhibit stronger generalization and interpretability. As shown in Figure 5, the relationship between engineering strain and hardness indicates that all material units of GS 316L stainless steel follow the strain hardening effect, which can be expressed as follows:

(1) $$\frac{\partial H}{\partial \varepsilon }>0$$

where $\varepsilon =\ln(\varepsilon_{e}+1)$ represents the true strain. The value of loss function inherently decreases and stabilizes as training progresses. Therefore, min(∂H/∂ε,0) can be used to characterize the strain hardening effect.

Strain, stress, tensile force, depth, and hardness have different dimensions, and their values differ by several orders. This would cause severe numerical issues in machine learning. Therefore, the aforementioned physical quantities were normalized to scale data with different features to the same range. Normalization of physical quantities can eliminate dimensional differences, accelerate model convergence, and enhance consistency in model performance between different features. Adding a horizontal line above the symbol indicates normalization. Based on the normalized physical quantities, the PINNs_εH loss function was defined as follows:

(2) $$L_{\varepsilon -d-H}=\underset{D_{\bar{U}}^{\mathrm{tr}}}{\mathrm{mean}}[\min(\partial \bar{H}/\partial \bar{\varepsilon },0){]}^{2}+\underset{D_{\bar{U}}^{\mathrm{tr}}}{\mathrm{mean}}{\left({\bar{H}}_{\exp}-\bar{H}\right)}^{2}$$

The second term in Equation (2) represents data-driven, where ${\bar{H}}_{\exp}$ is the measured hardness, and $\bar{H}$ is the hardness predicted by PINNs_εH. The dataset $D_{\bar{U}}$ consists of depth, true strain and hardness of GS 316L stainless steel. 80% of $D_{\bar{U}}$ was extracted through uniform random sampling to form the training set $D_{\bar{U}}^{tr}$, while the remaining 20% was used as the validation set $D_{\bar{U}}^{\mathrm{val}}$. The training set was used for the learning process of the PINNs model, helping it in extracting rules and features from the data. The validation set was employed to evaluate the performance of the PINNs model during training, aiding in the selection of optimal hyperparameters and preventing overfitting. The loss function of PINNs_εH was optimized using the adaptive learning rate mechanism of the Adaptive Moment Estimation (ADAM) optimizer with an initial learning rate of 0.001. A cosine decay strategy was implemented to periodically adjust the learning rate, mitigating early-stage oscillations and late-stage stagnation, with a minimum learning rate threshold of 1 × 10^−5^. Training proceeded for 3000 epochs with a batch size of 32. The training was terminated when the value of $L_{\varepsilon -d-H}$ was less than the preset error threshold to prevent overfitting.

The hyperbolic tangent function (tanh), which is smooth, continuous, and differentiable over its entire domain, was chosen as the activation function. The following operation was applied to each hidden layer:

(3) $${\bar{X}}_{i}=\tanh(W_{i}{\bar{X}}_{i-1}+b_{i})$$

where $W_{i}$ represents the weight matrix, $b_{i}$ denotes the bias vector, ${\bar{X}}_{i-1}$ is the input vector, and ${\bar{X}}_{i}$ is the output vector. The subscript *i* indicates the index of the hidden layer, ranging from 1 to *m*, where *m* is the total number of hidden layers. For given number of hidden layers and neurons, the parameters $W_{i}$ and $b_{i}$ of the PINNs_εH model can be obtained through training based on $D_{\bar{U}}^{\mathrm{tr}}$.

The performance of the neural network (NN) can be evaluated using the Mean Relative Error (*MRE*) and the coefficient of determination ($R^{2}$) of the output labels. When *MRE* is closer to 0 and $R^{2}$ is closer to 1, the performance of the NN is better. The MRE and $R^{2}$ are calculated as:

(4) $$MRE=\underset{D}{\mathrm{mean}}\left(\frac{\left|{\bar{X}}_{\exp}-\bar{X}\right|}{\left|{\bar{X}}_{\exp}\right|}\right)$$

and

(5) $$R^{2}=1-\frac{\underset{D}{\mathrm{mean}}{\left({\bar{X}}_{\exp}-\bar{X}\right)}^{2}}{\underset{D}{\mathrm{mean}}{\left[{\bar{X}}_{\exp}-\underset{D}{\mathrm{mean}}({\bar{X}}_{\exp})\right]}^{2}}$$

**Figure 7:**
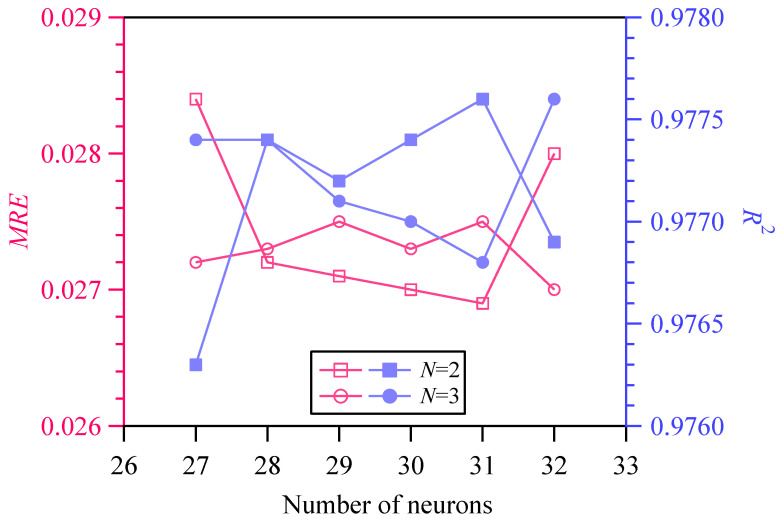
Performance evaluation of PINNs_εH architecture.

When the output label is hardness, ${\bar{X}}_{\exp}$ is assigned to ${\bar{H}}_{\exp}$, $\bar{X}$ is assigned to $\bar{H}$, respectively, and D represents the validation set $D_{\bar{U}}^{\mathrm{val}}$. Let the number of hidden layers be 2 and 3, and the number of neurons in each hidden layer be 27~32. Twelve network structures were constructed, and then twelve PINNs_εH models were trained based on $D_{\bar{U}}^{\mathrm{tr}}$ and evaluated by the validation set $D_{\bar{U}}^{\mathrm{val}}$. Substituting the predicted hardness from PINNs_εH models into Equations (4) and (5), the corresponding MRE and $R^{2}$ were calculated. It can be seen from Figure 7 that the NN with 2 hidden layers and 31 neurons per layer demonstrates the best performance. By the constructed PINNs_εH model, which is represented by the formula H=PINNs_εH(ε,d), a large amount of data on depth, true strain, and hardness can be obtained.

## 4. Depth-Dependent Constitutive Relationships of GS 316L Stainless Steel

### 4.1. Laminated Plate Model

**Figure 8:**
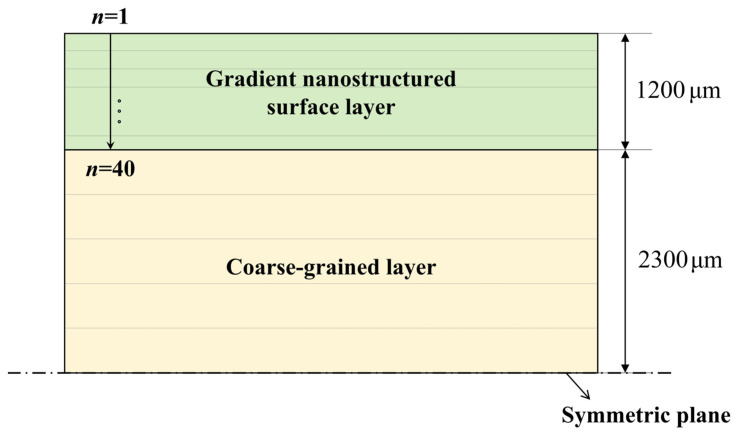
Laminated plate model of GS 316L plate specimen.

According to the microstructure characterization and hardness measurement results of GS 316L stainless steel plate, it can be seen that the two sides of the plate are GS surface layers with a thickness of 1200 μm, and the middle is a CG layer with a thickness of 4600 μm. The GS surface layer is symmetrical about the mid-plane of the plate. The GS surface layer can be regarded as a laminated plate composed of *n* thin layers, as illustrated in Figure 8. The macroscopic mechanical properties of each thin layer are uniform and isotropic. When *n* is large enough, the gradient variation characteristics of mechanical properties along the plate thickness direction can be well reproduced. Due to the symmetry of geometry and mechanical properties of the GS 316L plate about the mid-plane, a 1/2 symmetric model was used in the following.

The GS surface layer, with a thickness of 1200 μm, was divided into 40 thin layers whose thickness can be equal or unequal. The mechanical properties of the CG layer are uniform, so theoretically there is no need for layering. However, to facilitate the acquisition of stress–strain relationships at any depth, the CG layer was divided into five thin layers of equal thickness. The layering strategy based on laminated plate theory not only ensures high accuracy within the GS surface layer but also effectively saves computational resources. The initial depths of the mid-plane of each thin layer were extracted for the construction of datasets used in Section 4.2, Section 4.3 and Section 4.4. In order to improve the transfer efficiency, performance, and reliability of the PINNs model, the depth data in the pre-trained model and the transfer learning model must be consistent.

### 4.2. Depth-Dependent PINNs_Hσ Pre-Trained Model

**Figure 9:**
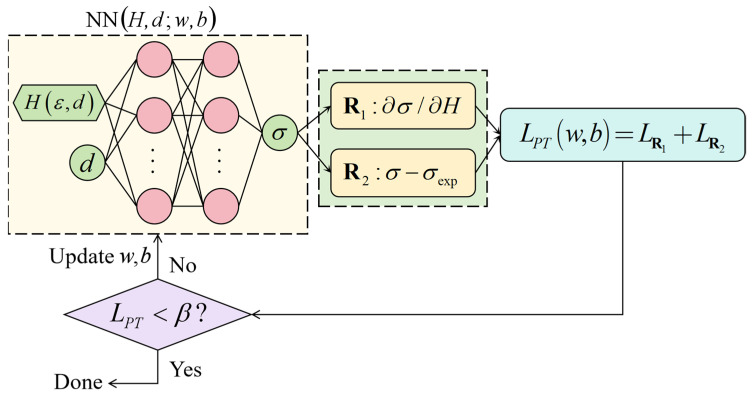
Constructing a depth-dependent PINNs_Hσ pre-trained model for hardness and true stress.

Based on the experimental results of CG 316L stainless steel, the PINNs_Hσ pre-trained model was constructed using a fully connected feedforward neural network, as shown in Figure 9. The input features are hardness *H* and initial depth *d*, and the output label is true stress *σ*. From the relationship curve of axial tensile load and true strain (Figure 4) and the distributions of hardness and engineering strain (Figure 5), a positive correlation between the true stress and hardness can be deduced. The positive correlation requires that the first derivative of true stress with respect to hardness must be greater than zero, i.e.:

(6) $$\frac{\partial \sigma }{\partial H}>0$$

To ensure that the PINNs_Hσ network satisfies Equation (6), the following loss function was defined:

(7) $$L_{PT}=\underset{D_{\bar{V}}^{\mathrm{tr}}}{\mathrm{mean}}{\left[\min(\partial \bar{\sigma }/\partial \bar{H},0)\right]}^{2}+\underset{D_{\bar{V}}^{\mathrm{tr}}}{\mathrm{mean}}{\left({\bar{\sigma }}_{\exp}-\bar{\sigma }\right)}^{2}$$

where $D_{\bar{V}}^{\mathrm{tr}}$ is the pre-training dataset, ${\bar{\sigma }}_{\exp}$ is the experimental results of true stress, and $\bar{\sigma }$ is the predicted true stress by PINNs_Hσ model. The dataset $D_{\bar{V}}^{\mathrm{tr}}$ consists of depth, hardness, and true stress of coarse-grained 316L stainless steel. The positive correlation between true stress and hardness is represented by the first term, and the data-driven is expressed by the second term. The hyperbolic tangent function was selected as the activation function, and the loss function was optimized by the ADAM optimizer with an initial learning rate of 0.005. A cosine decay strategy was implemented to periodically adjust the learning rate with a minimum learning rate threshold of 5 × 10^−5^. Training proceeded for 2000 epochs with a batch size of 16. The pre-training was terminated until the value of $L_{PT}$ was less than the preset error threshold.

**Figure 10:**
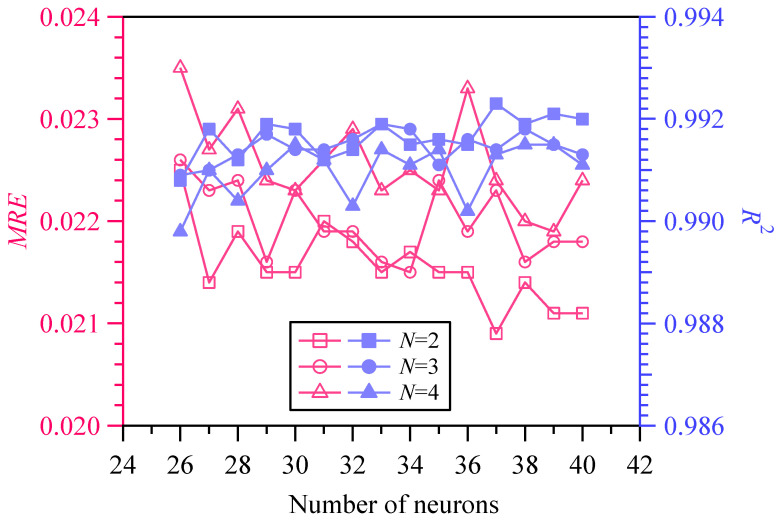
Performance evaluation of PINNs_Hσ architecture.

Let the number of hidden layers be 2, 3, and 4, and the number of neurons in each hidden layer be 26~40. Forty-five network architectures were then constructed. Substituting of the predicted true stress $\bar{\sigma }$ and the measured true stress ${\bar{\sigma }}_{\exp}$ into Equations (4) and (5), the values of MRE and $R^{2}$ were calculated. Comparing and analyzing MRE and $R^{2}$ shown in Figure 10, it can be concluded that the neural network with 2 hidden layers and 37 neurons has the best performance. The optimal network architecture evaluated by MRE and $R^{2}$ will be used for subsequent transfer learning.

### 4.3. Depth-Dependent PINNs_Hσ Transfer Learning Model

**Figure 11:**
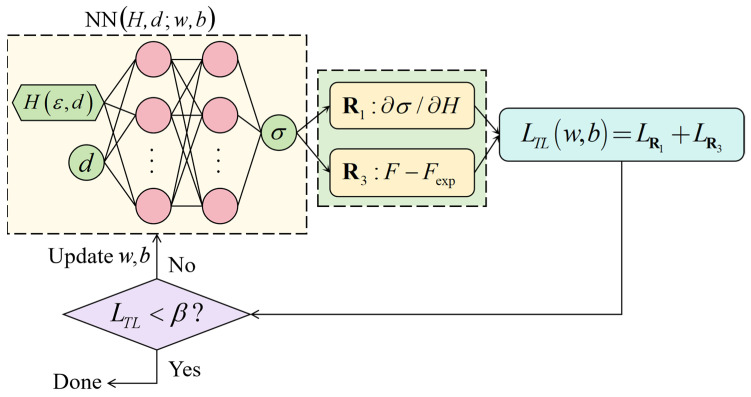
Constructing a depth-dependent PINNs_Hσ transfer learning model for hardness and true stress.

In order to ensure the continuity and consistency of feature mapping, the PINNs_Hσ transfer learning model has the same architecture as the PINNs_Hσ pre-trained model, which includes 2 hidden layers and 37 neurons, and adopts the tanh activation function, as shown in Figure 11. It should be noted that in the PINNs_Hσ transfer learning model, due to the lack of depth-dependent $\sigma_{\exp}$ data for GS 316L stainless steel, the physical constraint $R_{2}:\sigma -\sigma_{\exp}$ was replaced by $R_{3}:F-F_{\exp}$. The hardness-stress conversion weights obtained from the PINNs_Hσ pre-trained model were used as the initial values for the conversion weights in the transfer learning process.

The stress–strain responses of material units at different depths in GS materials are different from each other, making it impossible to obtain the related experimental data of stress, strain and hardness for each material unit through quasi-static tensile tests. The lack of stress data for GS materials makes transfer learning unable to perform data-driven computing like the pre-trained model. The overall stress–strain response of GS materials can be considered as the sum of the stress–strain responses of all material units. Therefore, the data-driven algorithm can be equivalently replaced by the following resultant force condition:

(8) $$F(\varepsilon )=\sum_{i=1}^{n}F_{i}(\varepsilon )$$

where F(ε) represents the measured axial tensile load, and $F_{i}(\varepsilon )$ denotes the axial load borne by material units at different depths. Furthermore, it is assumed that all material units of the GS materials still obey the positive correlation between stress and hardness, i.e., ∂σ/∂H>0. Therefore, the loss function of PINNs_Hσ transfer learning model can be defined as follows:

(9) $$L_{TL}=\underset{D_{\bar{\mathrm{TL}}}}{\mathrm{mean}}[\min(\partial \bar{\sigma }/\partial \bar{H},0){]}^{2}+\underset{D_{\bar{F}}}{\mathrm{mean}}{\left[{\bar{F}}_{\exp}(\varepsilon )-\bar{F}(\varepsilon )\right]}^{2}$$

where ${\bar{F}}_{\exp}(\varepsilon )$ is the measured axial tensile load, and $\bar{F}(\varepsilon )$ is the axial load predicted by PINNs_Hσ transfer learning model. The construction method of dataset $D_{\bar{\mathrm{TL}}}$ is as follows: firstly, 100 sets of true strain within the range of 0–40% were taken; secondly, 4500 sets of depth and true strain were obtained by cross-combining 100 strains and 45 depths; finally, 4500 sets of depth and hardness were calculated by PINNs_εH model. The construction method of dataset $D_{\bar{F}}$ with 100 sets of true strain and axial tensile load is as follows: 100 sets of true strain within the range of 0–40% were taken. The corresponding axial tensile loads with respect to the selected true strains were determined by using the experimental data shown in Figure 4. The loss function was optimized using the ADAM optimizer with an initial learning rate of 0.005, and transfer learning was terminated when $L_{TL}$ was less than the preset error threshold. By utilizing the PINNs_Hσ transfer learning model, σ=PINNs_Hσ(H,d), a vast amount of data on depth, hardness and true stress can be obtained.

### 4.4. Depth-Dependent PINNs_εσ Model

**Figure 12:**
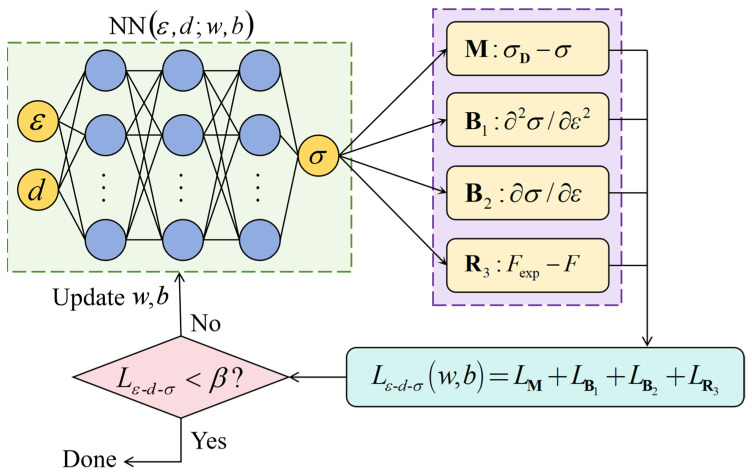
Constructing a depth-dependent PINNs_εσ model for true strain and true stress of GS materials.

By using hardness as an intermediate variable, the constitutive relationships between true strain and true stress can be established through the PINNs_εH model and the PINNs_Hσ model. A fully connected feedforward neural network was employed for the modeling PINNs_εσ, which has three hidden layers and each layer has 20 neurons, as shown in Figure 12. The input features are true strain *ε* and initial depth *d*, and the output label is true stress *σ*.

Through quasi-static uniaxial tensile tests, it is known that there is no obvious initial yield stage in the curves of true stress and true strain of both CG and GS 316L stainless steels. The linear deformation stage transits smoothly into the strain hardening stage. The curve of true stress and true strain is a monotonically increasing and convex smooth curve. Therefore, the monotonic increase in the curve of true stress and true strain can be expressed as follows:

(10) $$\frac{\partial \sigma }{\partial \varepsilon }>0$$

The convexity of the curve can be expressed as follows:

(11) $$\frac{\partial^{2}\sigma }{\partial \varepsilon^{2}}<0$$

The aforementioned physical constraints were characterized by $\max(\partial^{2}\sigma /\partial \varepsilon^{2},0)$ and min(∂σ/∂ε,0) in the loss function. During the elastic deformation, there is no strain hardening effect. The measured hardness of the elastic deformed specimen after unloading is the same as that of the undeformed specimen. Therefore, the method of obtaining stress based on hardness by machine learning is no longer applicable to the elastic deformation stage. The GS 316L stainless steel is an isotropic material, and the refinement of the microstructure has a negligible effect on both the elastic modulus and the Poisson’s ratio. Hooke’s law characterizing elastic stress and elastic strain of all material units should be identical, wherein the elastic modulus and Poisson’s ratio are 195 GPa and 0.3, respectively.

Considering the monotonic increasing and convex characteristics of the curve of true stress and true strain, as well as the uniform and isotropic mechanical properties during the linear elastic deformation stage, the following loss function was defined:

(12) $$\begin{array}{l} L_{\varepsilon -d-\sigma }=\underset{D_{\bar{W}}^{\mathrm{tr}}}{\mathrm{mean}}{\left({\bar{\sigma }}_{D}-\bar{\sigma }\right)}^{2}+\underset{D_{\bar{W}}^{\mathrm{tr}}}{\mathrm{mean}}[\max(\partial^{2}\bar{\sigma }/\partial {\bar{\varepsilon }}^{2},0){]}^{2}\\ \underset{D_{\bar{W}}^{\mathrm{tr}}}{+\mathrm{mean}}[\min(\partial \bar{\sigma }/\partial \bar{\varepsilon },0){]}^{2}+\underset{D_{\bar{P}}}{\mathrm{mean}}{\left[{\bar{F}}_{\exp}(\varepsilon )-\bar{F}(\varepsilon )\right]}^{2} \end{array}$$

where ${\bar{\sigma }}_{D}$ represents the true stress predicted by PINNs_Hσ transfer learning model, $\bar{\sigma }$ denotes the true stress predicted by PINNs_εσ model, $\bar{F}(\varepsilon )$ is the axial tensile load calculated from $\bar{\sigma }$, and ${\bar{F}}_{\exp}(\varepsilon )$ indicates the measured axial tensile load. The dataset $D_{\bar{W}}^{\mathrm{tr}}$ comprises associated depths, true strains, and true stresses. The dataset $D_{\bar{P}}$ consists of associated engineering strains and axial tensile loads.

The construction method of dataset $D_{\bar{W}}^{\mathrm{tr}}$ is as follows: (1) a certain number of true strains were taken within the range of 0–40%; (2) the true stresses were calculated using Hooke’s law; (3) the true stresses were also predicted by the PINNs_εH model and the PINNs_Hσ model; and (4) the true stress corresponding to the true strain is the smaller of the calculated and predicted values.

The construction method for dataset $D_{\bar{P}}$ is given as follows. For the linear elastic stage where the stress–strain relationship obeys Hooke’s law, and the strain-hardening stage where stress varies approximately linearly with strain, the intervals of true strain values were relatively large. In the initial yielding stage where stress varies non-linearly with strain, the intervals of true strain values are smaller. Based on the above strategy, a certain number of true strains were taken within the range of 0–40%, and the corresponding axial tensile loads were obtained from the curve of true strain and axial tensile load shown in Figure 4. Finally, 65 sets of true strain and axial tensile load were acquired.

**Figure 13:**
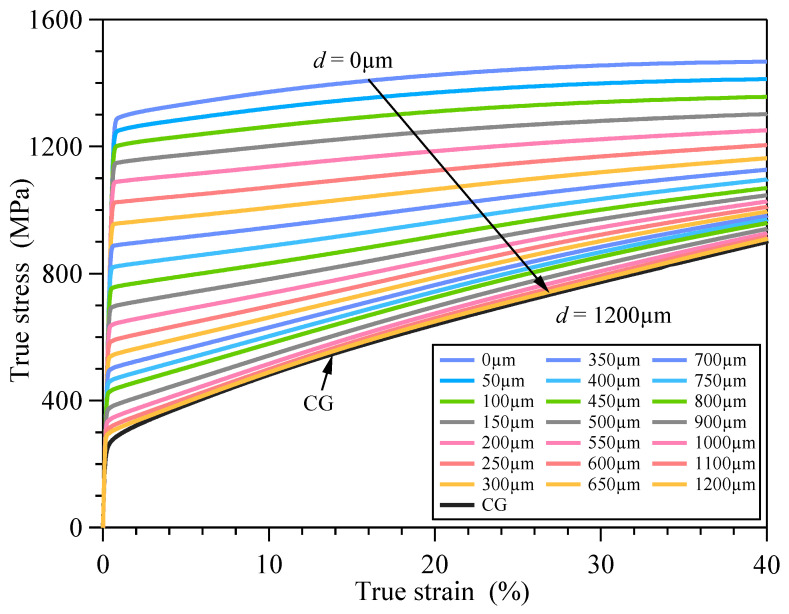
Elastic–plastic constitutive relationships of GS 316L stainless steel.

The PINNs_εσ loss function was optimized using the ADAM optimizer with an initial learning rate of 0.005. A cosine decay strategy was implemented to periodically adjust the learning rate with a minimum learning rate threshold of 5 × 10^−5^. Training proceeded for 100 epochs with a batch size of 256. The training was terminated when $L_{\varepsilon -d-\sigma }$ was less than the preset error threshold. By the σ=PINNs_εσ(ε,d) model, the depth-dependent constitutive relationships of GS 316L stainless steel were obtained. Figure 13 shows the elastic–plastic stress–strain responses of some material units corresponding to different depths from the surface. All material units exhibit strain hardening effects and the degree of strain hardening decreases with depth.

## 5. Validation of Elastic–Plastic Constitutive Relationships of GS 316L Stainless Steel

### 5.1. Three-Point Flexure Test

**Figure 14:**
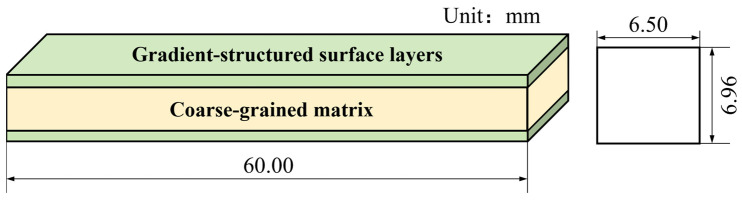
Three-point bending specimen.

The rectangular cross-section of the three-point bending specimen is shown in Figure 14. The length, width and height of the specimen are 60.00 mm, 6.50 mm, and 6.96 mm, respectively. The dimensions of the characteristic units of the microstructure vary gradually along the height of the specimen. The central region of the specimen is a CG matrix, and the upper and lower regions of the specimen are GS surface layers distributed symmetrically about the mid-plane.

**Figure 15:**
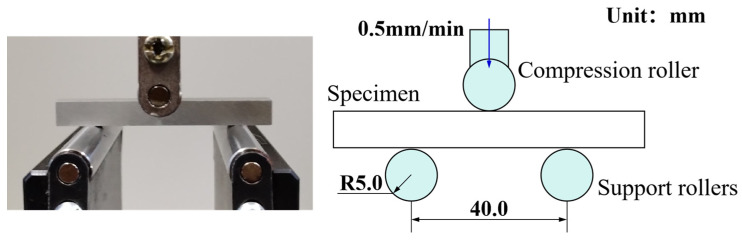
Experimental setup and schematic diagram of the three-point flexure test.

**Figure 16:**
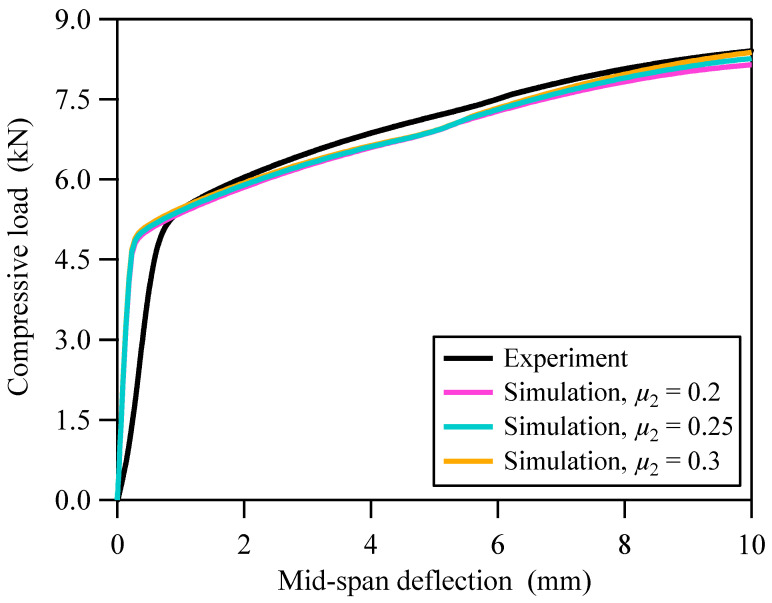
Experimental and predicted relationships between compressive load and mid-span deflection of three-point bending specimens.

Three-point flexure test was performed at room temperature using an Instron 3369 universal materials testing machine. The diameters of the compression roller and the two support rollers of the three-point flexure test fixture are 10.0 mm. The distance between the two support rollers is 40.0 mm. The specimen was symmetrically positioned around the two support rollers and the compression roller as shown in Figure 15. The lower support rollers were fully constrained, with the exception of the upper roller, which was moved 10.0 mm downwards at a constant speed of 0.5 mm/min. The specimen slid longitudinally relative to the support rollers during the test. No notable plastic deformation was observed in the contact area of the flexed specimen. The measured curve of the compressive load and mid-span deflection of the three-point bending specimen is shown in Figure 16.

### 5.2. Finite Element Simulation

**Figure 17:**
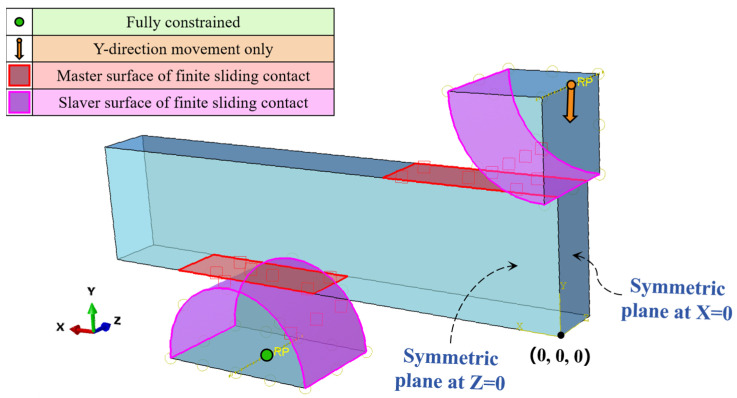
Finite element model of three-point flexure test.

Based on the symmetry of the length and width directions of the three-point bending specimen, as well as the symmetry of the displacement and force boundary conditions, a 1/4 finite element model was developed as shown in Figure 17. The right side (*x* = 0) and the front side (*z* = 0) of the specimen are the symmetry planes, and the symmetric displacement constraints in the *x*- and *z*-directions were applied, respectively. The possible contact regions between the compression roller and the specimen, and between the support roller and the specimen, were set as a hard contact in the normal direction and a finite sliding contact with friction in the tangential direction. The coefficient of friction between the compression roller and the specimen ($\mu_{1}$) was taken as a fixed value of 0.3. On the other hand, the coefficients of friction in the contact area between the support roller and the specimen ($\mu_{2}$) were taken as 0.2, 0.25, and 0.3 in order to study the effect of the coefficient of friction on the relationship between deflection and compressive load. Due to the very high rigidity and low deformation of the support roller and the compression roller, they were setup as rigid bodies. Based on the actual working condition of the fixture in the three-point flexure test, the lower support roller was fully constrained, and the compression roller was only allowed to move in the *y*-direction.

**Figure 18:**
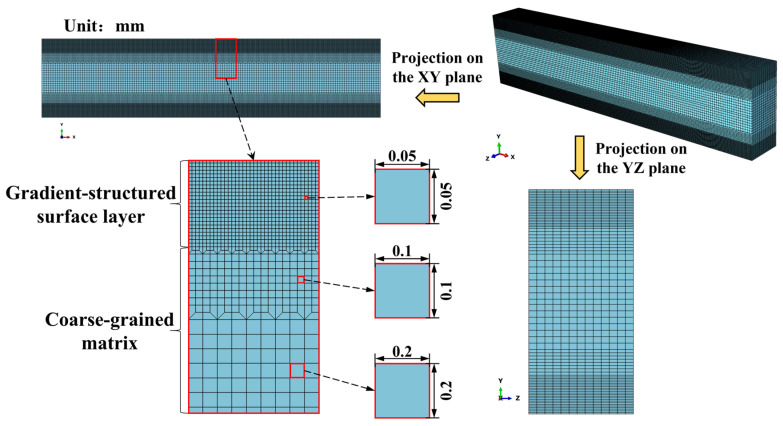
Finite element meshes of the three-point bending specimen.

Finite element simulation of the three-point flexure test was carried out using the general-purpose software ABAQUS (version [2022]). The three-point bending specimen was modeled by a hexahedral element (C3D8), and the support roller and the compression roller were modeled using a reduced integration hexahedral element (C3D8R). Based on the gradient changes in mechanical properties and of the bending normal stress along the height direction of the three-point bending specimen, a strategy of gradient variation in mesh size was used in order to effectively balance computational efficiency and simulation, as shown in Figure 18. The projection of the finest mesh onto the *xy*-plane is a square with sides of 0.05 mm, increasing to 0.2 mm by two transition levels. All elements of the three-point bending specimen have a dimension of 0.25 mm in the *z*-direction.

**Figure 19:**
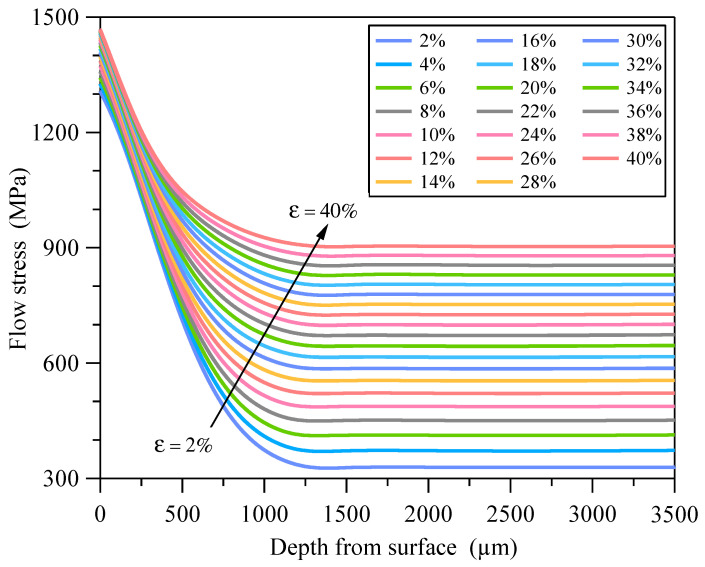
Variation curves of flow stress with depth at different tensile strains.

Based on the σ=PINNs_εσ(ε,d) model the flow stress versus depth curve of GS 316L stainless steel at a given strain can be obtained, as shown in Figure 19. The mesh size in the GS surface layer is 0.05 mm, and accordingly the maximum depth difference at different locations in the same element is 0.05 mm. The difference in flow stress caused by this depth difference is less than 5%. Therefore, assuming that the macroscopic mechanical properties of the material are the same in the one element, the elastic–plastic constitutive relationships of the material can be obtained by the σ=PINNs_εσ(ε,d) model corresponding to the centroid of the finite element mesh. The GS 316L stainless steel has an elastic modulus of 195 GPa and a Poisson’s ratio of 0.3.

### 5.3. Results and Discussion

The predicted curve of the compressive load and mid-span deflection of the three-point bending specimen is shown in Figure 16. The pink, blue, and orange curves correspond to the predicted results when the coefficients of friction of the contact area between the support roller and the specimen are 0.2, 0.25, and 0.3, respectively. It can be seen that, for the same deflection, an increase in the coefficient of friction results in a subsequent increase in the compressive load.

At the initial stage of compression (deflection between 0.0 mm and 0.8 mm), the compressive load obtained from the three-point flexure test is lower than that obtained from the finite element simulation. The initial clearances between the equipment, fixtures, and the specimen reduced the system’s effective stiffness during initial loading, leading to a non-linear relationship between load and deflection. Therefore, this discrepancy can be attributed to the initial clearances between the equipment, fixtures, and the specimen. When the deflection exceeds 0.8 mm, the gap effect disappears, and the experimental data are in good agreement with the numerical results. When the downward deflection of the compression roller is 10 mm, the maximum true strain of the specimen from the finite element simulation is about 41.5%. The accuracy of the constitutive model, σ=PINNs_εσ(ε,d) of GS 316L stainless steel is effectively verified.

## 6. Conclusions

The depth-dependent elastic–plastic constitutive relationships of GS 316L stainless steel were determined using PINNs. Conventional testing fails to capture the stress–strain response of individual material units within the heterogeneous GS layer. Leveraging the inherent strain hardening of 316L and the positive correlation between hardness, strain, and stress, hardness served as a key measurable intermediate variable.

A depth-dependent PINNs_εH model was established using directly measured hardness–strain–tension data from GS specimens. For stress prediction, a PINNs_Hσ model was first pre-trained on uniform CG 316L data. This pre-trained model was then adapted to GS materials via transfer learning, utilizing equivalent static force conditions as physical constraints instead of direct stress data. Combining these models via hardness, depth-dependent strain–stress data were generated. Depth-dependent strain–stress data of GS 316L stainless steel were obtained from the PINNs_εH model and the PINNs_Hσ transfer learning model, with hardness as an intermediate variable. Based on the loss function incorporating the physical constraint of no apparent yield limit as well as Hooke’s law of linear elasticity, the PINNs_εσ model characterizing the elastic–plastic constitutive relationships of GS 316L stainless steel was constructed by strain–stress data.

Validation through three-point flexure tests and corresponding finite element simulations demonstrated excellent agreement in load-deflection curves, confirming the PINNs_εσ model accurately characterizes the depth-dependent stress–strain response of GS 316L stainless steel from elastic deformation to the onset of necking. This methodology, leveraging hardness as a measurable intermediate variable, provides a viable approach for determining position-dependent constitutive relationships in heterogeneous materials. It circumvents the difficulties associated with fabricating miniature specimens for direct mechanical testing at specific depths. Furthermore, the approach effectively accounts for the material’s strain hardening effect and enables the resulting model to predict stress–strain responses at arbitrary depths. However, the applicability of the PINNs_εσ model is confined to the uniform deformation regime. The model does not currently capture constitutive behavior during necking or fracture processes, which limits its utility in failure analyses involving localized deformation.

## Data Availability

The original contributions presented in the study are included in the article, further inquiries can be directed to the corresponding author.
